# Surgical and orthodontic rapid palatal expansion in adults using a modified palatal partial osteotomy technique (ppot): Technique description and clinical experience

**DOI:** 10.4317/jced.56313

**Published:** 2020-06-01

**Authors:** Massimo Galli, Francesca-Romana Federici, Dario Di Nardo, Marco Yusef, Antonio Morese, Rebecca-Jewel Manenti, Luca Testarelli

**Affiliations:** 1 MD. Department of Oral and Maxillo-Facial Sciences, Sapienza University of Rome, Italy; 2 DDS. Department of Oral and Maxillo-Facial Sciences, Sapienza University of Rome, Italy; 3 DDS, PhD. Department of Oral and Maxillo-Facial Sciences, Sapienza University of Rome, Italy; 4 MD, DDS. Department of Oral and Maxillo-Facial Sciences, Sapienza University of Rome, Italy

## Abstract

Transversal hypoplasia of the upper maxilla is a frequent condition between malocclusions. The rapid maxillary expansion (RME) is an already consolidated technique for these types of defects. This case report analyzes the outcome of a novel surgical technique that we named TOPP (Partial Palatal osteotomy technique) aiming to provide scientifically proven data over the percentage of relapse and the long-term stability of this type of surgical assisted palatal expansion. A 24 year old male patient with a hyperdivergent class III, presenting the absence of 1.1 due to a teenage trauma and a transversal contraction of the upper arch was selected for the surgery. The mucoperiosteal flap was performed at a palatal level with a paramarginal arch shape (from region 1.4 to 2.4) due to preserve the nasal-incisal vascular bundle and the mucoperiosteum was detached from the floor of the nose. A horizontal osteotomy was performed at 4-5 mm above the roots apexes; a sagittal osteotomy in a posterior direction was done at the level of the midline to divide the mesiopalatine suture and separate the maxilla in two halves. The only bony attachment that remained was represented by the perpendicular lamina of the palatal bone. The TOPP technique showed that it is possible to have a better control of both the intercanine and intermolar expansion, that is more difficult in the case of a conventional SARME. Other goals were a greater view and access to the site and the reduction of the risk of damaging the palatine fibromucosa. The incision of the archform paramarginal flap improves certainly the conditions of the palatal fibromucosa in the post operative and allows the reduction of the soft tissues’ elastic return.

** Key words:**Rapid maxillary expansion, orthognatic surgery, maxillary osteotomy.

## Introduction

Transversal hypoplasia of the upper maxilla is a frequent condition between malocclusions. The rapid maxillary expansion (RME) is an already consolidated technique for these types of defects.

Even though it’s the treatment of choice for growing teenagers, in patients that have reached skeletal maturity instead, the RME has demonstrated to have limited orthopedic effects for the maxillary skeletal structures due to the greater thickness of the bones and due to the welding of the maxillary sutures. These aspects lead to a greater risk of relapse, more pain, dental tipping with possible severe damages to the periodontal surrounding structures (1).

To avoid these problems, the SARME technique has been used to achieve an orthopedic expansion of the jaws. SARME consists in the surgically assisted rapid expansion associated with appliances for the bone-borne jaw expansion that are able to limit the appearance of these undesired effects (2). The anatomical structures that are considered to be involved in the resistance of the maxillary expansions are the median palatine suture, the zygomatic-maxillary suture, the zygomatic-temporal suture and the zygomatic frontal suture (3). The areas of resistance to the expansion of the jaws are mostly considered to be the piriform opening (anteriorly), the maxillary tuberosity (laterally), the pterygomaxillary junction (posteriorly) and the median palatine suture (mesially) (4).

This case report analyzes the outcome of a novel technique that we named TOPP (Partial Palatal osteotomy technique) aiming to provide scientifically proven data over the percentage of relapse and the long-term stability of this type of surgical assisted palatal expansion by reducing the elastic return of the palatal fibromucosa.

## Case Report

**Figure 1:**
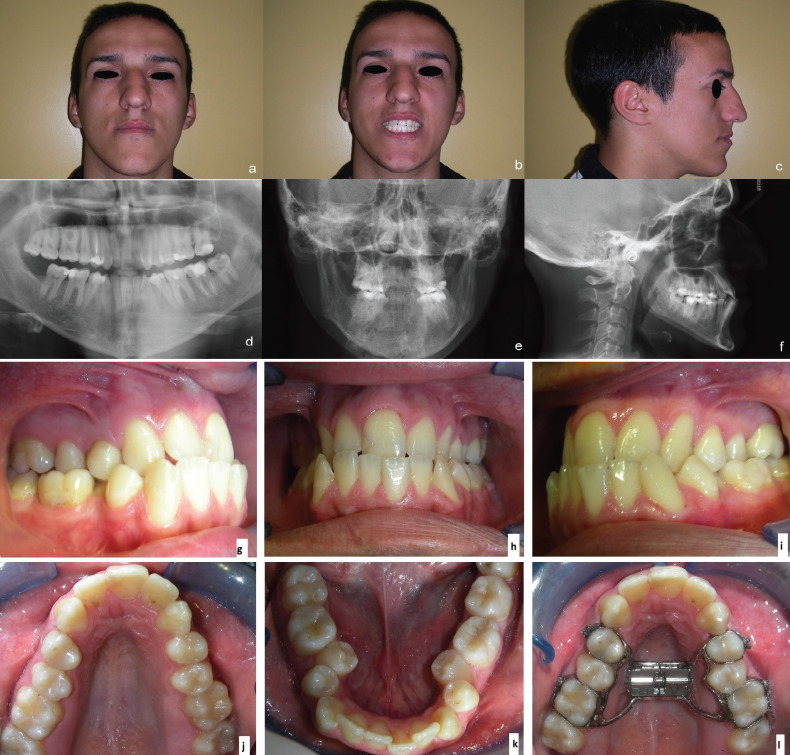
Initial pictures of the patient (a-c). Panoramic radiograph (d). Posterior-anterior radiograph (e). Latero-lateral radiograph (f). Right lateral view (g), frontal view (h), left lateral view (i), palatal view (j), lingual view (k), expander appliance cemented on first premolars and molars (l).

A 24 years old male patient with a hyperdivergent class III, presenting the absence of 1.1 due to a teenage trauma and a transversal contraction of the upper arch (Fig. 1). At the medical history examination, the patient did not referred any systemic pathologies or particular conditions. The procedure was approved by the institutional review board of the private institute where the surgery was made and the risk assessment was in according with the Declaration of Helsinki.

**Figure 2:**
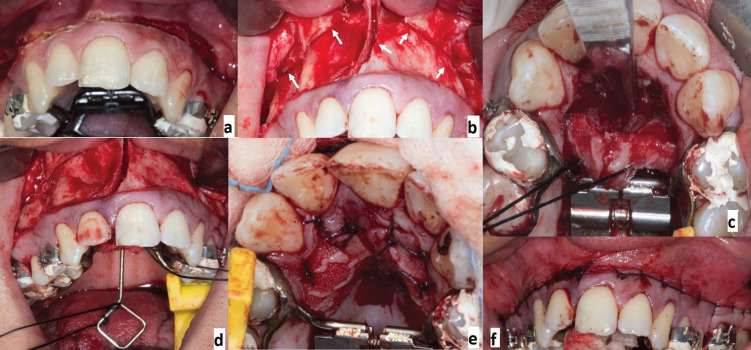
Vestibular flap incision (a), medial and lateral osteotomy (white arrows) (b), palatal view of the paramarginal archform flap with sagittal osteotomy (c), activation of the expander (5\6 intraoperative turns) (d), frontal view of the continuing vestibular suture (e), palatal view of the discontinuing suture of the palatal fibromucosa (f).

The surgical intervention (Fig. 2) required general and local infiltrative anesthesia with vasoconstrictor at the buccal and palatal mucosa. After performing a high circumvestibular incision starting from the region of 1.6 up to the region of the 2.6, the soft tissues were detached to skeletonize the maxilla; the mucoperiosteal flap was performed at a palatal level with a paramarginal arch shape (from region 1.4 to 2.4) due to preserve the nasal-incisal vascular bundle. Subsequently, the mucoperiosteum was detached from the floor of the nose. A horizontal osteotomy was performed at 4-5 mm above the roots apexes, starting from the piriform opening to reach posteriorly the maxillary tuberosity in correspondence of the pterygoid lamine. The disjunction was performed by using a blunt osteotome between the osteocartilaginous part of the nasal septum and the floor of the nasal fossa; afterwards, a sagittal osteotomy in a posterior direction was done at the level of the midline to divide the mesiopalatine suture and separate the maxilla in two halves. The only bony attachment that remains is represented by the perpendicular lamina of the palatal bone. Before the repositioning of the flaps and suture, the mobility of the bone segments was checked through the activation of the maxillary expander appliance placed in position.

The activation through the expander was of 5 or 6 complete turns, which formed a millimetrically evident diastema. The surgery was finalized with the irrigation of the surgical site and the suture of the soft tissues.

Antibiotics, corticosteroids and analgesics were prescribed to reduce the risk of post-operative complications and to facilitate tissue healing. Indications to not eat any hard food and not rip off anything with teeth were given to the patient, aiming to not solicitate the sites of the osteotomy, in order to promote the formation of new bone instead of fibrous tissue that could colonize the space between the bone segments.

**Figure 3:**
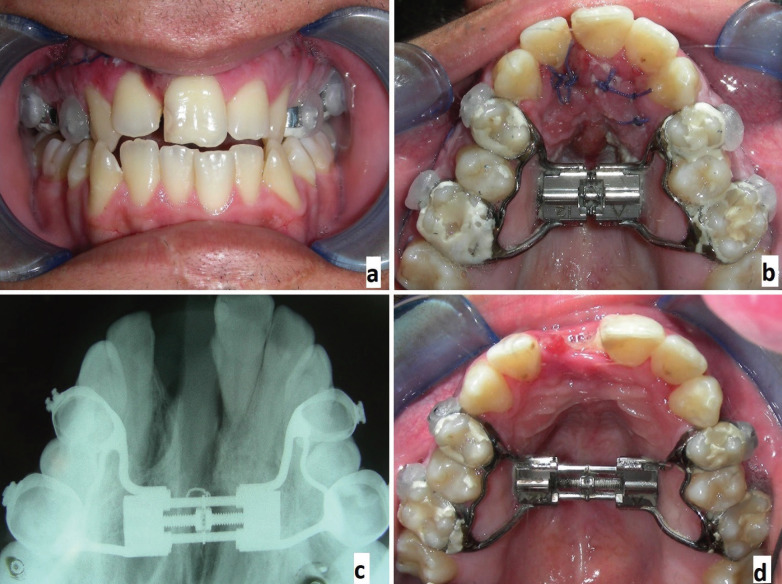
Frontal view one week from the surgery; diastema between elements 1.1 and 1.2 (a), palatal view of the healing of the palatal fibromucosa (b), occlusal radigraph two weeks from the surgery (c), palatal view after two weeks from the surgery (d).

Because it was expected to find a significant resistance in the areas of the osteotomies after 4-6 weeks from the surgery, the expansion was performed during this range of time. The patient completed the expansion under precise instructions, by activating the device after a post-surgical latency period of 5 or 6 days, with 2\4 of turns per day, twice a day, for a maximum of 4/4 turns per day (1 mm), for a period of 7-15 days. The entity of the expansion depended on the amount of pain perceived by the patient: he was prescribed to not overturn the device if the perceived pain was more than moderate. After this period, the expander device was modified with a resin block that covered the screw and then kept for a period of 6 months as a retainer, to allow the consolidation of the bone structures (Fig. 3). In some situations, it is advised to build a new retainer device that should be used for other 2 or 3 months to ensure the stability of the expansion performed.

## Discussion

The stability of the surgically assisted maxillary expansion has been widely examined with discouraging results by Krebs, Stickfish (5), Linder-Aronson and Lindgren (6). Moussa (7) and others have encountered a good stability in 55 patients after 8 to 10 years post retention. Mew (8) hasn’t registered in his study any significant relapses after 2-6 years post retention in 25 patients treated with intermolar expanders. Kuo and Will (9) refer in their treated cases with surgically assisted expansion, that 13 patients out of 15 have maintained completely their original expansion.

By analyzing a group of patients that undergone a SARME procedure, Jeffrey L. Berger (10) has demonstrated that, depending on the aspect that we consider from the surgery, different values of relapses have been shown. For these reasons, some strategies to avoid the relapse have been proposed: an initial hypercorrection (not accepted by all, for example Krebs) (11) or an adequate post operative retention to apply towards the end of the orthodontic treatment for at least the first year after the surgical intervention (12).

In relation to the segmental osteotomy technique, the most complete data collection is found in a study made by Phillips *et al.* (13), where it is shown that the majority of the expansion is usually obtained in the second molar region; this is ineviTable in case of two segments osteotomy and it may occur also in case of multiple segmentations. In the canine area instead, both less expansion and less relapse occurr. SARME is also familiar with many complications like paresthesia of the infraorbital nerve, intraoperative bleeding of the pterygoid plexus, aseptic necrosis of the interincisal papilla with bone sequestrum, light deviation of the bone septum (14).

The TOPP technique, that presents a palatal approach, including both an arch-form paramarginal shaped flap and an osteotomy done from below, allows to eliminate the first cause of relapse, that is the resistance of the palatal fibromucosa.

The intercanine width, that increased surgically of 4,84 mm, after one year and the removal of the orthodontic appliance have decreased of 1,2 mm. The intermolar width instead, after an increase of 5,78 mm, has decreased after one year of 1,01 mm. The interalveolar width, augmented of 3,42 mm through expansion, after one year showed a decrease of 0,14 mm. Moreover, the width of the maxilla and the zygomatic bone that have increased respectively of 3,00 mm and 0,53 mm after one year from the surgery, show that the jaw expansion has decreased of 2,48 mm, while the zygomatic bone remained sTable in that position.

Regarding the TOPP technique, the already exiguous follow-up doesn’t allow us to provide certain data on the long-term stability, but the approach attempts to avoid the principal cause of relapse and this represents a promising rationale.

This technique has been introduced to guarantee benefits in respect to the SARME conventional technique and to diminish some inconvenient results, by giving the correct importance to the middle palatine suture. The TOPP technique showed that it is possible to have a better control of both the intercanine and intermolar expansion, that is more difficult in the case of a conventional SARME, in which the intermolar width results limited.

Other goals of this modified technique are a greater view and access to the site, the reduction in the danger of damaging the palatine fibromucosa from the inside through an accurate planning of the design of the incisional flap and a minor risk of creating an oronasal communication, that is often present in the SARME.

In the traditional SARME technique the activation of the expander leads to a notable stress concentration in the palatal fibromucosa (that is inevitable when using a technique that doesn’t contemplate palatal flaps), due to the tension produced from the appliance itself. Performing a flap also on the palatal side, the tensile stress is reduced. (4)

The danger of the double access surgery is linked also to an alteration of the vascularization that could be overcome through a careful study of the design of the incision. (15) Keeping into account the anatomy and distribution of the vessels and by performing a good detachment of the flap, lacerations could be avoided and an adequate suture could lead, when possible, to a first intention healing.

The variation of the technique was conceived with the objective to improve the traditional SARME technique in terms of better visibility and access but also to try and diminish the percentage of post-treatment relapse and to increase the long term stability.

The incision of the archform paramarginal flap improves certainly the conditions of the palatal fibromucosa in the post operative and allows the reduction of the first cause of relapse in this kind of intervention, which is made by the soft tissues’ elastic return.
